# Erratum to: Pharmacology of anticoagulants used in the treatment of venous thromboembolism

**DOI:** 10.1007/s11239-016-1363-2

**Published:** 2016-05-04

**Authors:** Edith A. Nutescu, Allison Burnett, John Fanikos, Sarah Spinler, Ann Wittkowsky

**Affiliations:** 1 Department of Pharmacy Systems Outcomes and Policy and Center for Pharmacoepidemiology & Pharmacoeconomic Research, College of Pharmacy, University of Illinois at Chicago, Chicago, IL USA; 2 Inpatient Antithrombosis Services, University of New Mexico Hospital, University of New Mexico College of Pharmacy, Albuquerque, NM USA; 3 Brigham and Women’s Hospital, Massachusetts College of Pharmacy, Boston, MA USA; 4 Philadelphia College of Pharmacy and Science, Philadelphia, PA USA; 5 University of Washington School of Pharmacy, Seattle, WA USA

**Keywords:** Pharmacology, Mechanism of action, Anticoagulants, Warfarin, Heparins, Direct oral anticoagulants (DOAC)

## Abstract

Anticoagulant drugs are the foundation of therapy for patients with VTE. While effective therapeutic agents, anticoagulants can also result in hemorrhage and other side effects. Thus, anticoagulant therapy selection should be guided by the risks, benefits and pharmacologic characteristics of each agent for each patient. Safe use of anticoagulants requires not only an in-depth knowledge of their pharmacologic properties but also a comprehensive approach to patient management and education. This paper will summarize the key pharmacologic properties of the anticoagulant agents used in the treatment of patients with VTE.

## Introduction

Anticoagulant drugs are the mainstay of therapy for patients with venous thromboembolism (VTE). Specific treatment decisions are guided by balancing the risks and benefits of various anticoagulants. The treatment of VTE can be divided into 3 phases: acute (first 5–10 days), long-term (first 3 months), and extended (beyond 3 months) [1]. The acute treatment phase of VTE consists of administering a rapid-onset parenteral anticoagulant [unfractionated heparin (UFH), low molecular weight heparin (LMWH), fondaparinux] or direct oral anticoagulant (DOAC; apixaban, rivaroxaban). Long-term and extended phase anticoagulation for VTE is usually accomplished using oral anticoagulant agents such as warfarin, or one of the DOACs (apixaban, dabigatran, edoxaban and rivaroxaban) [1, 2]. The optimal selection and management of anticoagulant drugs for the treatment of VTE requires not only an in-depth knowledge of the efficacy, safety and clinical outcomes data but also of the pharmacology for each agent. This paper will summarize the key pharmacologic properties of the anticoagulant agents used in the treatment of VTE.

### Unfractionated heparin

UFHs are naturally-occurring glycosaminoglycans derived from porcine intestinal or bovine lung mucosal tissues [3–6]. Commercial UFH is composed of a heterogeneous group of highly sulfated polysaccharide chains varying in molecular weight from 3000 to 30,000 daltons (mean 15,000 daltons) or approximately 45 saccharide units [3–7]. They are considered indirect anticoagulants because their activity requires the presence of antithrombin (AT), an endogenous anticoagulant glycoprotein produced by the liver. Approximately one-third of heparin chains contain an active pentasaccharide sequence capable of binding to AT (Fig. 1). This heparin-AT complex inhibits thrombin (factor IIa) and factors Xa, IXa, XIa, and XIIa. In order to inhibit thrombin activity, an UFH chain has to bind to both AT and thrombin simultaneously to form a ternary complex (UFH-AT-thrombin complex). In contrast, in order to inhibit Factor-Xa activity, UFH only needs to form a binary complex by binding to AT. Thus, in order to catalyze thrombin inhibition, UFH chains need to be longer than 18 saccharide units, whereas chains that are shorter than 18 saccharide units can still catalyze Factor-Xa inhibition. In both cases however, binding to AT occurs at the active pentasaccharide sequence level. UFH exhibits equal inhibitory activity against factor-Xa and thrombin, binding these in a 1:1 ratio. Once UFH binds and activates AT, it can readily dissociate and bind to additional AT, providing a continuous anticoagulant effect. UFH has no fibrinolytic activity and therefore does not dissolve an existing thrombus, but does prevent its propagation and growth. UFH blocks thrombin-induced activation of factors V and VII, enhances tissue factor pathway inhibitor (TFPI) release by vascular endothelial cells reducing the procoagulant activity of the tissue factor-VIIa complex and at higher concentrations catalyzes thrombin inhibition through heparin cofactor II (HCII) [3, 4, 7]. UFH is also known to inhibit tumor growth as well as a variety of protease enzymes including myosin TPase, RNA-dependent DNA polymerase, elastase, and renin [7].

**Fig. 1:**
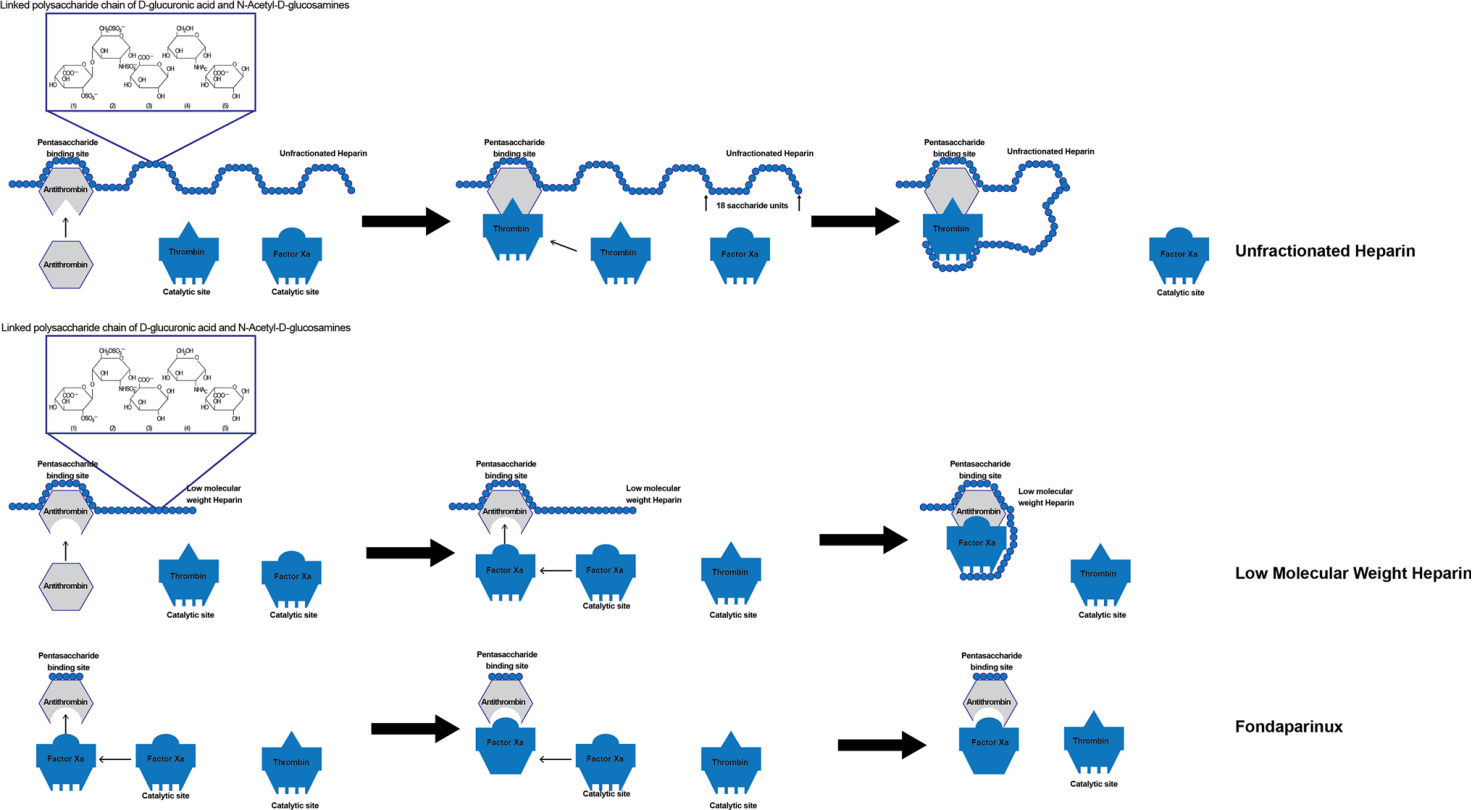
Mechanism of action of heparin, low molecular weight heparin, and pentasaccharide (fondaparinux)

After entering the blood stream UFH binds to plasma proteins which contribute to its low bioavailability and variable anticoagulant response [3–7]. As UFH is poorly absorbed orally, intravenous (IV) infusion or subcutaneous (SC) injection are the preferred routes of administration [8]. IV administration (with a bolus dose) rapidly achieves therapeutic plasma concentrations and is the preferred method of administration when rapid anticoagulation is required. When given SC, the bioavailability of UFH ranges from 30 to 70 %, depending on the dose given. Therefore, higher doses (>30,000 units/day) of UFH must be given if the SC route of administration is used to deliver therapeutic doses of the agent. The onset of anticoagulation is delayed by 1–2 h if UFH is given by SC injection whereas the onset is immediate (seconds to minutes) if given IV. The half-life of UFH is dose dependent and ranges from 30 to 90 min but may be significantly longer, up to 150 min, with high doses.

UFH’s elimination from the systemic circulation is dose-related and occurs through two independent mechanisms [3, 4]. In the initial phase, enzymatic degradation occurs via a rapid, saturable zero-order process. The second phase is a slower, non-saturable, renal-mediated first-order process. Lower UFH doses are primarily cleared via enzymatic processes, whereas higher doses are primarily renally eliminated. At therapeutic doses, UFH is cleared primarily in the initial phase with the higher molecular weight chains being cleared more rapidly than lower weight counterparts. As clearance becomes dependent on renal function, increased or prolonged UFH dosing provides a disproportionate increase in both the intensity and the duration of the anticoagulant effect. Patients with active thrombosis may require higher UFH doses due to a more rapid elimination or variations in the plasma concentrations of heparin-binding proteins.

Due to interpatient variability in dose response and changes in patient response over time, UFH requires monitoring and dosing adjustments. Since plasma UFH levels can’t be measured directly, the anticoagulant response to IV UFH administration is monitored using the activated partial thromboplastin time (aPTT) [3]. The aPTT is a measure of the time (in seconds) it takes for thrombus formation (from the activation of factor XII within the intrinsic pathway to the last step of fibrin formation in the common pathway). The aPTT may be influenced by laboratory reagent sensitivity, monitoring equipment, variability in plasma proteins and circulating clotting factors. Traditionally, the therapeutic aPTT range was defined as 1.5–2.5 times the control aPTT value. However, due to changes in reagents and instrumentation over time, as well as variations in the reagents and instruments across laboratories, each institution should establish their own therapeutic range for UFH. The institution-specific therapeutic aPTT range should correlate with a plasma heparin concentration of 0.2–0.4 units/mL by protamine titration or 0.3–0.7 units/mL by an amidolytic antifactor Xa assay [6, 9]. An aPTT should be obtained at baseline and 6 h after initiating the heparin infusion as this time is required to reach steady-state. The aPTT should subsequently be measured every 6 h (including after each dose change) and adjusted per an institution-specific nomogram/protocol until two sequential therapeutic aPTTs are achieved. Then, monitoring may be decreased to once daily. The UFH dose is then adjusted based on the aPTT measurement and the institutional-specific therapeutic range. Alternatively anti-Factor-Xa level monitoring (or the heparin assay) may be used, which does not depend on thromboplastin reagents, is insensitive to plasma proteins, and may improve monitoring outcomes [10]. In patients with heparin resistance, and those with baseline elevated aPTT due to antiphospholipid antibodies, anti-Factor Xa concentrations may be a more accurate method of monitoring the patient’s response to heparin.

The dose of UFH required to achieve a therapeutic anticoagulant response is correlated to the patient’s weight [4, 11]. Thus, to optimize UFH delivery and attain the therapeutic threshold quickly, weight based dosing nomograms are recommended for the acute treatment of thromboembolic disease. Weight-based dosing nomograms have been associated with significantly higher initial heparin doses, shorter time to therapeutic aPTTs and no increase in bleeding events. Heparin dosing nomograms will differ from hospital to hospital due to differences in thromboplastin reagents and inter-laboratory standardizations in aPTT measurements [9, 10].

The major complications of UFH therapy include bleeding (major bleeding, 0 to 5 %; fatal bleeding, 0 to 3 %), heparin-induced thrombocytopenia (1–5 %), and osteoporosis (2–3 %) [11, 12]. Hypersensitivity reactions, alopecia and hyperkalemia have also been reported but are more rare side effects [13, 14]. Hemorrhagic episodes are associated with the intensity and stability of anticoagulation, route of administration, and concomitant use of antiplatelet or fibrinolytic therapy [11, 12, 15, 16, 17]. Patient- specific risk factors are the most important consideration when determining the bleeding risk and include age, gender, history of previous bleeding, renal function, body weight, risk of falls or trauma, recent surgery and alcohol consumption [13].

The treatment of severe UFH related bleeding includes reversal of anticoagulant effect with protamine sulfate, transfusion therapy, and supportive care [18, 19]. Protamine sulfate is a cationic protein that binds to UFH, forming a stable salt and terminating its anticoagulant action. Protamine dosing is dependent on timing of the last heparin dose. For immediate reversal (<30 min since the last heparin dose), 1 mg of protamine is administered for every 100 units of heparin [20]. A follow up aPTT can be used to evaluate the reversal response. When UFH is given as a continuous IV infusion, only UFH delivered during the preceding 2–2.5 h should be included in the calculation to determine the protamine dose. If the dose of heparin is unknown, the maximal tolerated protamine dose of 50 mg can be administered as a slow IV infusion over 10 min followed by serial measurements of aPTT. The effects of UFH are neutralized in 5 min, and the effects of protamine persist for 2 h. If the bleeding is not controlled or the anticoagulant effect rebounds, repeated doses of protamine may be administered [13].

Severe adverse reactions to protamine, such as hypotension and bradycardia, are common. Reaction severity may be reduced by slowing the administration time of protamine over 1-3 min (maximum administration rate should not exceed 5 mg per min). Allergic responses and the development of antiprotamine antibodies are more common in patients who have been previously exposed to the drug for UFH neutralization. Patients at risk of developing antiprotamine antibodies can be pretreated with corticosteroid and antihistamine medications.

Heparin-induced thrombocytopenia without (HIT) or with thrombosis (HITT) is an immune-mediated disorder that results from antibodies being formed against the heparin–platelet factor 4 complex [21–24]. The incidence of HITT in critically ill patients ranges from 1 to 5 % and is associated with the development of thrombocytopenia and life-threatening thrombosis in approximately 30–50 % of cases [21–24]. This immune-mediate response typically occurs in patients exposed to UFH for 5–7 days, or within 24 h if the patient had recent previous heparin exposure. A 50 % reduction in platelet count from baseline occurring 4–10 days after UFH initiation or formation of a new thrombus during UFH or LMWH therapy should raise suspicion for HIT [20]. In all patients on therapeutic heparin, platelet count should be measured prior to the initiation of UFH and at least every other day for the first 4–10 days of therapy. The incidence of HIT is approximately one-tenth lower with LMWH than with UFH [21]. However, LMWH cannot be used in the setting of HIT, nor should it be used in suspected HIT due to cross reactivity between glycosaminoglycans. Direct thrombin inhibitors are the treatment of choice for patients with HIT and HITT [22–24].

Patients receiving heparin for periods of more than 1 month are also at an increased risk for osteoporosis and development of vertebral fractures (approximately 2 % incidence). Osteoporosis reportedly occurs less frequently in patients treated with LMWHs as compared to UFH, and it is typically associated with long-term therapy [3].

### Low molecular weight heparins

The LMWHs are derived by chemical or enzymatic depolymerization of UFH, with isolation and extraction of low molecular weight fragments. Like UFH, LMWHs prevent the propagation and growth of formed thrombi, but do not break down existing clots [14]. Two LMWHs are currently available in the United States: dalteparin and enoxaparin. Also like UFH, LMWH are indirect anticoagulants exerting their anticoagulant effect by binding to AT through a specific pentasaccharide sequence (Fig. 1). The primary difference in the pharmacologic activity of UFH and LMWH is their relative inhibition of thrombin (factor-IIa) and factor-Xa. Smaller heparin fragments cannot bind AT and thrombin simultaneously. Due to their smaller chain length and molecular weight (4500–5000 Daltons), LMWHs have relatively greater activity against factor-Xa and inhibit thrombin to a lesser degree. The antifactor Xa-to-IIa activity ratio for the LMWHs ranges from 2:1 to 4:1 [25].

Compared with UFH, LMWHs have improved pharmacodynamic and pharmacokinetic properties, a more predictable anticoagulant response and a more favorable side-effect profile. Consequently, routine monitoring of anticoagulation activity and dose adjustments are not required in most patients. The bioavailability of LMWHs following subcutaneous injection approaches 100 %. Peak anti-Factor-Xa activity occurs about 3–4 h following a subcutaneous dose. Unlike UFH, the pharmacokinetics of LMWHs are linear across doses studied [26].

Enoxaparin and dalteparin are metabolized in the liver by desulfation and/or depolymerization to lower molecular weight fragments with reduced biologic activity. About 3 % of an active dose of dalteparin and 10 % of enoxaparin is eliminated renally as active fragments. Compared to UFH, LMWHs are more dependent upon renal clearance. The elimination half-lifes of dalteparin and enoxaparin are approximately 3–4.5 h following a single dose (Table 1). Following repeated doses in healthy subjects, the half-life of dalteparin is about 5 h, and for enoxaparin is roughly 7 h. The apparent volume of distribution of LMWHs as measured by anti-Factor-Xa activity, approaches blood volume [26, 27]. The half-lives of enoxaparin and dalteparin increase in patients with chronic kidney disease as a result of reduced clearance, and accumulation is expected unless doses are reduced [28, 29]. There is a strong association with creatinine clearance (CrCl) and clearance of anti-Factor-Xa activity with enoxaparin [13]. The mean half-life in dialysis patients was 5.7 h following a single intravenous dose of dalteparin [29]. A twofold increase in AUC was reported following a single intravenous enoxaparin dose in patients receiving hemodialysis [28]. Little data are available in obese patients but anti-Factor-Xa levels appear to be in the expected range on a dose per kg total body weight basis for doses administered in patients weighing up to 144 kg with enoxaparin and 190 kg with dalteparin [13].

**Table 1:** Comparison of the pharmacologic features of heparin and its derivatives

Feature	Heparin	LMWH	Fondaparinux
Source	Biological	Biological	Synthetic
Molecular weight (Da)	15000	5000	1500
Target	XIIa, IXa, XIa, Xa and IIa	Xa > IIa	Xa
Bioavailability (%)^a^	30	90	100
Half-life (h)	1	4	17
Monitoring test	aPTT, Anti-Factor-Xa	Anti-Factor-Xa	Anti-Factor-Xa
Renal excretion	No	Yes	Yes
Antidote	Protamine	Protamine	None
Incidence of HIT (%)	<5	<1	Unreported

*Da* Dalton, *h* hours, *HIT* heparin-induced thrombocytopenia, *LMWH* low molecular weight heparin

^a^Following subcutaneous injection

Because LMWHs are a mixture of longer and shorter glycosaminoglycan fragments, serum concentrations of drug are not measureable. Therefore, the pharmacokinetics of LMWHs are determined based upon anticoagulation activity measured by a calibrated anti-Factor-Xa assay. Routine monitoring of LMWHs is not recommended in the majority of patients. However, the anticoagulant effect of LMWHs may be measured using anti-Factor-Xa levels in certain high-risk situations such as patients with chronic kidney disease, severe obesity, pregnancy and in children [30]. Monitoring of trough anti-Factor-Xa levels, taken just prior to the next dose, can be considered to assess accumulation in patients with renal impairment receiving prophylactic or treatment doses of LMWHs. A maximum trough anti-Factor-Xa level is considered to be 0.5 IU/mL. The role of measuring peak anti-Factor-Xa levels in LMWH patients is less clear as it has not been correlated to clinical outcomes. Peak levels of enoxaparin, drawn 4 h post-dose, following twice daily subcutaneous administration for treatment of VTE have been reported in the range of 0.6–1.0 IU/mL. Following once daily administration of dalteparin or enoxaparin for VTE treatment, the observed peak anti-Factor-Xa concentration is 1.0–2.0 IU/mL. Peak anti-Factor-Xa levels of enoxaparin observed in patients with acute coronary syndromes are 0.5–1.20 IU/mL. The clinical significance of elevated anti -Factor Xa levels are unknown, and there is no suggested dose reduction to achieve a reduced anti-Factor-Xa level [30]. LMWHs may increase the aPTT and ACT to a variable degree. Thus, these assays are not suitable for monitoring LMWH anticoagulant activity. Enoxaparin administration may prolong the aPTT by up to 20 s whereas there is a more pronounced effect following dalteparin administration [28, 31]. There are limited reports suggesting that at higher doses, the aPTT correlates with dalteparin anti-Factor-Xa activity [32, 33].

Similar to UFH, bleeding is the major complication associated with LMWHs. The incidence of major bleeding reported in clinical trials is less than 3 % [10, 11]. Minor bleeding, especially bruising at the injection site, occurs frequently. Protamine sulfate will partially reverse the anticoagulant effects of the LMWHs and should be administered in the event of major bleeding. Due to its limited binding to LMWH chains, protamine only neutralizes about 60 % of LMWH anticoagulant activity. If LMWH needs to be reversed and has been administered within the previous 8 h, it is suggested to give 1 mg protamine sulfate per 1 mg of enoxaparin or 100 anti-Factor-Xa units of dalteparin [13]. If the bleeding is not controlled, it is recommended to give 0.5 mg of protamine sulfate for every 100 anti-Factor-Xa units of LMWH and to use smaller protamine doses if more than 8 h have lapsed since the last LMWH dose.

LMWHs have less interaction with the heparin binding proteins platelet factor 4, protamine, lipase, and histidine-rich glycoprotein, and consequently are associated with a lower rate of HIT compared to UFH [13]. However, LMWHs cross-react with heparin antibodies in vitro and should not be given as an alternative anticoagulant in patients with a diagnosis or history of HIT. Platelet counts should be monitored every few days during the first 2 weeks of therapeutic LMWH use and periodically thereafter.

### Fondaparinux

Fondaparinux is a synthetic analog of the pentasaccharide sequence found within heparin chains and is a specific inhibitor of activated Factor-Xa. Like LMWHs and UFH, fondaparinux is indirect-acting and must first bind to AT to exert its anticoagulant activity (Fig. 1). Due to its small size, fondaparinux exerts inhibitory activity specifically against factor-Xa and has no effect on thrombin.

Administered subcutaneously, fondaparinux has 100 % bioavailability and is distributed into blood volume. Peak fondaparinux levels occur 2–3 h following subcutaneous administration [34, 35]. Fondaparinux is eliminated renally as unchanged drug with a half-life of 17–21 h in healthy subjects with normal renal function (Table 1). Thus, the anticoagulant effect of fondaparinux will persist for 2–4 days after stopping the drug and even longer in patients with renal impairment. The half-life of fondaparinux is prolonged and the AUC increased in elderly patients and those with chronic kidney disease or acute kidney injury. The total clearance of fondaparinux is reduced in patients with CrCl less than 80 mL/min and is about 55 % lower in patients with CrCl less than 30 mL/min compared to patients without renal impairment. No dosage adjustment is recommended for Child-Pugh Category B hepatic impairment. The pharmacokinetics of fondaparinux are not significantly different in females versus males or in healthy Asians versus white subjects. In patients weighing less than 50 kg, the total clearance of fondaparinux is reduced by 30 % [35].

Routine coagulation monitoring for fondaparinux is not recommended. However, anti-Factor-Xa activity following fondaparinux injection can be measured using an appropriate chromogenic-based anti-Factor-Xa assay that has been calibrated using fondaparinux. Anti-Factor-Xa assays that have been calibrated using an UFH or LMWH standards are not appropriate for fondaparinux. Mean peak and trough levels observed following prophylactic doses average 0.39–0.50 and 0.14–0.19 mg/L, respectively. Following therapeutic doses, observed mean peaks and troughs are 1.20–1.26 and 0.46–0.62 mg/dL, respectively. For treatment of VTE, peaks and troughs are similar across all body weights using recommended dosing [35]. Similar to LMWH, it should be emphasized that fondaparinux anti-Factor-Xa levels have not been correlated with clinical outcomes.

Anticoagulant activity of fondaparinux can also be measured using the prothrombinase-induced clotting time [36]. Peak levels are observed at 3 h post-dose. Elevated fondaparinux concentrations may prolong the PT. Some aPTT assays may be prolonged following prophylactic and therapeutic fondaparinux doses while up to 50 % of aPTT assays may be prolonged with elevated fondaparinux concentrations [37].

As fondaparinux is not metabolized in the liver it has few drug interactions [35, 38]. However, concurrent use with other antithrombotic agents increases the risk of bleeding. Unlike the heparins, fondaparinux does not affect platelet function and does not react with the heparin platelet factor (PF)-4 antibodies seen in patients with HIT. Thus it has a theoretical role in treatment and prevention of HIT/HITTS, and may be a preferred parenteral anticoagulant in patients with a history of HIT.

As with other anticoagulants, the major side effect associated with fondaparinux is bleeding. There currently is no specific antidote for fondaparinux and it is not reversed by protamine [38]. In the event of major bleeding, fresh-frozen plasma and/or factor concentrates may be considered. However, as factor concentrates carry a risk of thrombosis and can have a significant financial impact on care, judicious use is imperative [35, 38].

### Warfarin

Warfarin is a vitamin K antagonist that interferes with the hepatic synthesis of the procoagulant vitamin K dependent clotting factors II, VII, IX and X, as well as the synthesis of the anticoagulant proteins C, S and Z [39] (Fig. 2). These clotting factors are derived from precursor compounds and become biologically active through gamma-carboxylation of glutamic acid residues at the NH2-terminal molecular region. Gamma-carboxylation requires the presence of vitamin KH2, a reduced form of vitamin K that is oxidized to vitamin KO, an inactive form of vitamin K. The action of vitamin K epoxide reductase (VKOR) converts vitamin KO to vitamin K, followed by transformation to vitamin KH2 by vitamin K1 reductase. Through this vitamin K hepatic recycling process, a continuous supply of vitamin KH2 is available for clotting factor synthesis. By inhibiting both VKOR and vitamin K1 reductase, warfarin causes an accumulation of biologically inactive vitamin KO. This mechanism effectively reduces the hepatic synthesis of vitamin K dependent clotting factors, as well as proteins C, S and Z [40].

**Fig. 2:**
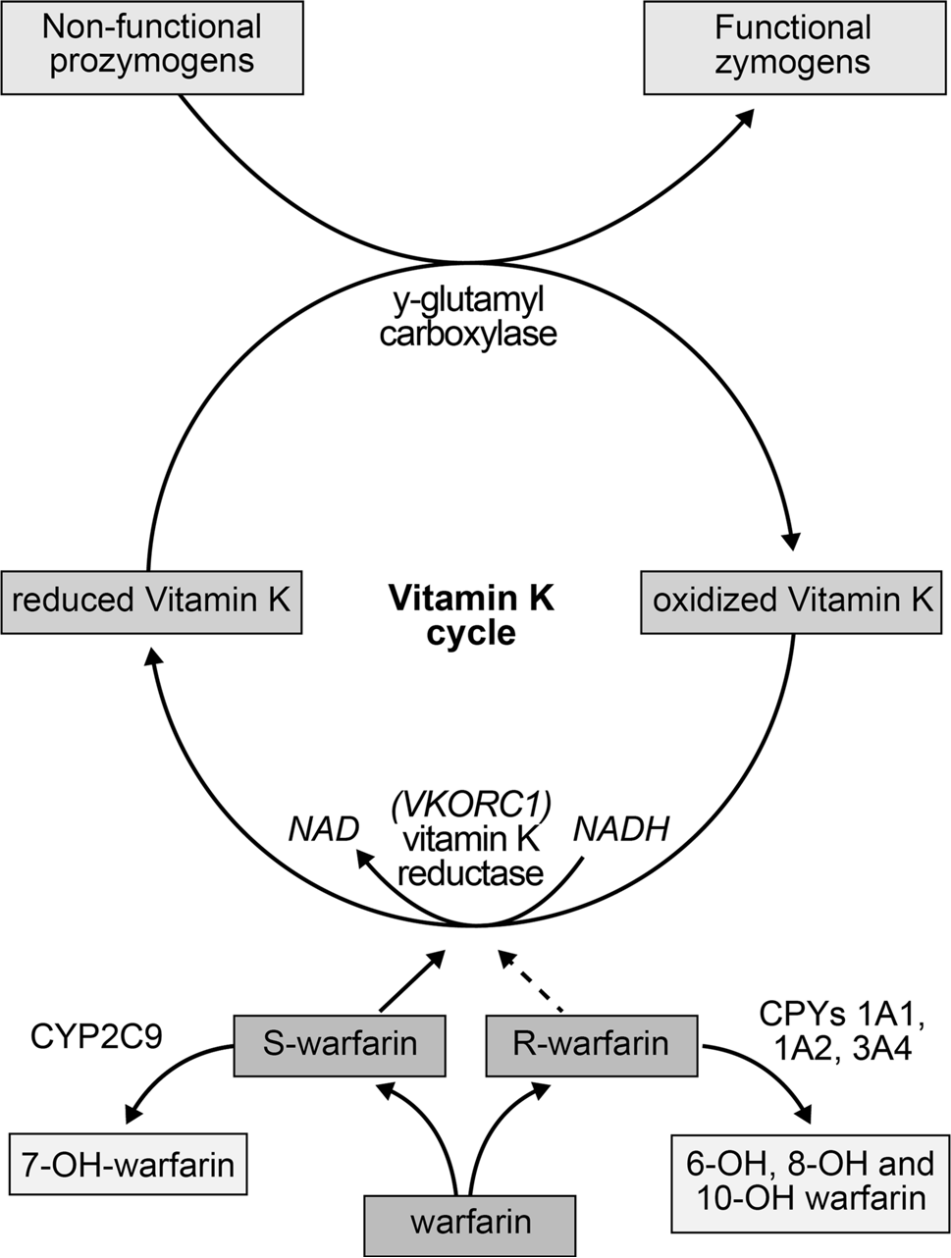
Pharmacology and mechanism of action of warfarin

Warfarin has no effect on circulating coagulation factors that have been previously formed. Thus, its anticoagulant effects are not apparent until the activated vitamin K dependent clotting factors are depleted. The timing of this depletion is dependent on the biologic half-life of each clotting factor (Table 2) [41]. As a result, the full anticoagulant effect of warfarin does not occur for at least 3–7 days after initiating oral administration or making a change in warfarin dose. The natural anticoagulant proteins C, S and Z are inhibited more rapidly and reductions in their concentration before the clotting factors are depleted can lead to a paradoxical hypercoagulable state during the first few days of warfarin therapy. It is for this reason that patients with acute thrombosis should receive a fast-acting anticoagulant (heparin, LMWH, or fondaparinux) while transitioning to warfarin therapy. When warfarin therapy is discontinued, vitamin K dependent clotting factors and anticoagulant proteins gradually return to pre-treatment concentrations.

**Table 2:** Half-lives of vitamin K-dependent proteins

Factor	Half-life (h)
II	42–72
VII	4–6
IX	21–30
X	27–48
Protein C	8
Protein S Protein Z	60 40–45

Warfarin is a racemic mixture of R and S enantiomers, optical isomers that display significant differences in pharmacokinetic and pharmacodynamic properties (Table 3) [42]. As a result of differences in receptor affinity to vitamin K reductase enzymes, S-warfarin is 2.7–3.8 more potent than R-warfarin. While the bioavailability, volume of distribution and protein binding of R- and S-warfarin are similar, their stereoselective metabolism and elimination half-lives differ significantly [43]. S-warfarin is approximately 90 % oxidized to inactive metabolites, primarily by CYP2C9 to a lesser extent by CYP3A4. Reduction to diasteriomeric alcohols accounts for the remainder of S-warfarin metabolism. In comparison, R-warfarin is approximately 60 % oxidized by CYP1A2, CYP3A4 and CYP2C19 to inactive metabolites and 40 % reduced to alcohol derivatives. The inactive oxidative metabolites and reduced alcohol derivatives of warfarin are eliminated by urinary excretion [44].

**Table 3:** Pharmacokinetic and pharmacodynamic properties of warfarin enantiomers

	R-warfarin	S-warfarin
Bioavailability	95–100 %	95–100 %
Volume of distribution	0.12–0.22 L/kg	0.11–0.19 L/kg
Protein binding	98.7–99.9 %	98.9–100 %
Elimination half-life	45 h (20–70 h)	29 h (18–52 h)
Hepatic metabolism	40 % reduction 60 % oxidation 1A2 > 3A4 > 2C19	10 % reduction 90 % oxidation 2C9 > 3A4
Stereospecific potency	1.0 (reference)	2.7–3.8 × R warfarin

Variations in patient genotype have been shown to affect warfarin dose requirements [45]. Specifically, the VKORC1 and CYP2C9 genotypes explain about 10 to 45 % of the overall warfarin dose variance [39, 45]. The CYP2C9 Arg144Cys (*2) and Ile359Leu (*3) polymorphisms reduce warfarin clearance and dose requirements and increase the risk of warfarin-related bleeding [46, 47]. The CYP2C9 Asp360Glu (*5), 10601delA (*6), Arg150His (*8) and Arg335Trp (*11) alleles occur predominately in African–Americans and also reduce dose requirements [48]. The VKORC1-1639G>A variant increases sensitivity to warfarin, thus leading to lower warfarin dose requirements [49].

Several algorithms that incorporate CYP2C9 genotype and VKORC1 haplotype along with other patient characteristics to predict warfarin maintenance dosing requirements have been developed and showed efficacy in better predicting warfarin stable doses when compared to clinical algorithms. Based on these data, the FDA recommends incorporating patient’s genotype information in guiding warfarin dosing when such information is available [45]. However, randomized studies to date showed mixed results of the impact of pharmacogenomic-based dosing on clinical and health utilization outcomes. Therefore, pharmacogenomic-based warfarin dosing has not yet been widely adopted in clinical practice and some guidelines recommend against routine ordering of genetic testing [39].

Warfarin has a narrow therapeutic index, requiring frequent dose adjustments. In addition to hepatic metabolism and genotype, warfarin dose requirements are influenced by diet, drug–drug interactions, and health status. Therefore, warfarin dose must be determined by frequent laboratory monitoring. The therapeutic effect of warfarin is monitored by the prothrombin time (PT), expressed as international normalized ratio (INR) [50]. The PT is sensitive to changes in serum concentrations of the vitamin K dependent clotting factors. By adding calcium and a tissue thromboplastin to plasma collected by venipuncture, activation of the extrinsic pathway of the clotting cascade is accelerated, and time to clot formation is measured in seconds [51].

Thromboplastin reagents used for PT monitoring display considerable variability in their ability to detect the clotting defect induced by warfarin. To standardize test results, the World Health Organization developed a system by which each commercial reagent batch produced by any manufacturer is assigned an International Sensitivity Index (ISI) that describes its comparison to an international reference thromboplastin, which has an ISI of 1.0 [52]. The ISI is used to mathematically convert prothrombin time in seconds to the INR, using the formula:

$$ {\text{INR = }}\left( {\frac{{{\text{PT}}_{\text{patient}} }}{{{\text{PT}}_{{{\text{mean}}\,{\text{normal}}}} }}} \right)^{\text{ISI}} $$

Using this method, PT results obtained at different laboratories are generally consistent as long as the instrumentation on which PT will be measured is also calibrated appropriately [53, 54]. A number of point-of-care devices have been developed that use whole blood obtained by fingerstick for rapid measurement of INR [55]. These test systems allow for rapid availability of test results and can be used for patient self-testing at home. The goal or target INR for each patient is based on the indication for warfarin therapy. For the treatment of VTE, the INR target is 2.5 with an acceptable range of 2–3.

Warfarin is highly susceptible to interactions with prescription and non-prescription drugs, as well as with herbal and other natural products [56]. Concurrent use of agents that alter the absorption, distribution, metabolism or excretion of warfarin can result in pharmacokinetic interactions that may elevate or reduce the INR, increasing the risk of hemorrhagic or thromboembolic complications, respectively. In addition, pharmacodynamic interactions can influence the response to warfarin without altering its pharmacokinetics, or increase the risk of bleeding or thromboembolism without influencing the INR (Table 4).

**Table 4:** Examples of warfarin drug interactions by mechanism and effect on INR

Category	Mechanism	Effect	Common examples
Pharmacodynamic interactions	Increased synthesis of clotting factors	Decrease INR	Vitamin K
	Decreased synthesis of clotting factors	Increase INR	Cephalosporins
	Reduced catabolism of clotting factors	Decrease INR	Methimazole Propylthiouracil
	Increased catabolism of clotting factors	Increase INR	Thyroid hormones
	Impaired vitamin K production by gut flora	Increase INR	Aminoglycosides Tetracyclines
	Additive anticoagulant response	Increase bleeding risk without influencing INR	Anticoagulants
	Concurrent antiplatelet therapy	Increase bleeding risk without influencing INR	Antiplatelet agents
Pharmacokinetic interactions	Induction of warfarin metabolism	Decrease INR	Barbiturates Carbamazepine Nafcillin Rifampin
	Reduced absorption of warfarin	Decrease INR	Cholestyramine Colestipol
	Inhibition of warfarin metabolism	Increase INR	Amiodarone Azole antifungals Fluoroquinolone Antibiotics Macrolide antibiotics Metronidazole Sulfa antibiotics

In patients taking warfarin, significant interactions may occur when interacting drugs are initiated, or discontinued, or when there is a change in the dose of the interacting drug. However, patient susceptibility to drug interactions is highly variable. The magnitude of the response, the time of onset and the duration of the interaction are influenced by patient characteristics, including pharmacogenomics, that themselves influence clotting factor synthesis and degradation, as well as the pharmacokinetics and pharmacodynamics of both the interacting drug and the R and S enantiomers of warfarin [57, 58].

A multitude of disease states and patient conditions influence sensitivity to warfarin. These conditions should be considered during initiation of therapy when the starting dose of warfarin needs to be determined, as well as later in therapy when onset, exacerbation, or improvement in these conditions may alter maintenance dosing requirements for warfarin (Table 5).

**Table 5:** Conditions that may influence response to warfarin

Advanced age
Alcohol use
Chewing tobacco
Cigarette smoking
Clinical congestive heart failure
Diarrhea
Dietary vitamin K intake
Fever
Following heart valve replacement
Hemodialysis
Hepatic disease
Hypoalbuminemia
Nutritional status
Pregnancy/lactation
Renal disease
Thyroid disease

As with other anticoagulants, warfarin’s primary side effect is bleeding [39, 45]. The annual incidence of major bleeding ranges from 1 to 10 % and bleeding risk is associated with the intensity and stability of anticoagulation therapy. Higher INRs result in higher bleeding risk. Vitamin K may be used to reverse warfarin’s effect in cases of major bleeding and/or warfarin over-anticoagulation. Vitamin K can be given by IV or oral route; the SC route is not recommended. When given SC, vitamin K is erratically absorbed and frequently ineffective. The IV route is reserved for cases of severe warfarin overdose and when patients are actively bleeding. Anaphylactoid reactions have been reported with rapid IV administration; therefore, slow infusion is recommended. An oral dose of vitamin K will reduce INR within 24 h. If INR is still elevated after 24 h, another dose of oral vitamin K can be given. The dose of vitamin K should be based on the degree of INR elevation and whether bleeding is present. A dose of 2 to 2.5 mg given orally is recommended when INR is greater than 10 and there is no active bleeding, while a higher dose 5-10 mg given via slow IV is recommended in cases when bleeding is present. Higher doses (e.g., 10 mg) can lead to prolonged warfarin resistance. In cases of life-threatening bleeding, fresh-frozen plasma or clotting factor concentrates should be administered, in addition to IV vitamin K. In patients in whom INR is less than 10 and there is no active bleeding or imminent risk of bleeding, simply withholding warfarin until INR decreases to within therapeutic range and reducing the weekly dose with more frequent monitoring is appropriate [14, 39, 40, 45].

Two other side effects associated with warfarin that providers should be aware of, warfarin induced skin necrosis and purple toe syndrome, are rare but potentially severe. [14, 39, 40, 45]. Warfarin-induced skin necrosis presents as an eggplant-colored skin lesion or a maculopapular rash that can progress to necrotic gangrene. It usually manifests in fatty areas such as the abdomen, buttocks, and breasts. The incidence is less than 0.1 %, and it generally appears during the first week of therapy. Risk factors include protein C or S deficiency and large loading doses of warfarin. The mechanism is thought to be due to imbalances between procoagulant and anticoagulant proteins early in the course of warfarin therapy. In warfarin-induced purple toe syndrome patients present with a purplish discoloration of their toes, typically within the first few weeks of therapy. If either of these side effects are suspected, warfarin therapy should be discontinued immediately and an alternative anticoagulant given.

### Direct oral anticoagulants

Currently, four direct oral anticoagulants (DOACs; apixaban, dabigatran, edoxaban, rivaroxaban) are commercially available in the United States and are approved for the treatment of VTE [59–62] (Table 6). The DOACs are direct anticoagulants with intrinsic anticoagulant activity and do not require binding to AT or other cofactors to exert their effect. They are small molecules (~400–600 daltons), able to penetrate and bind both clot-bound and free-floating thrombin [63]. Each of the DOACs inhibit a serine protease single target within the common pathway of the coagulation cascade during the final stages of clot formation (Fig. 3). This specificity provides a linear dose response, wide therapeutic index, allows for fixed dosing and precludes the need for routine monitoring of the anticoagulant effect of these agents [39, 64]. As with all anticoagulants, the major adverse effect of DOACs is bleeding. There are currently no specific antidotes for the apixaban, edoxaban and rivaroxaban, although several are in advanced stages of development. A specific antidote, idarucizumab, has recently been approved by the FDA for the reversal of dabigatran. In the event of a major haemorrhage and if a specific antidote is lacking, prothrombin complex concentrates or recombinant factor VIIa may be considered if the patient is refractory to standard approaches; however, their efficacy has not been clearly established. Concurrent use of DOACs with thrombolytics, NSAIDs or antiplatelet agents may significantly increase bleeding complications, and should be avoided whenever possible [59–62]. It is important to note that quantitative plasma concentration thresholds beyond which a patient would be at increased risk of clotting or bleeding have not been established for any of DOACs, and routine monitoring is not recommended [65–67].

**Table 6:** Comparative pharmacokinetics and pharmacodynamics of oral anticoagulants

	Warfarin	Dabigatran	Rivaroxaban	Apixaban	Edoxaban
Target(s)	IIa, VIIa, IXa, Xa	IIa	Xa	Xa	Xa
Prodrug	No	Yes	No	No	No
Bioavailability (%)	80–100	6.5 (pH dependent)	80	50	62
Volume of distribution (L)	10	50–70	50	23	>300
Peak effect	4–5 days	1.5–3 h	2–4 h	1–3 h	1–2 h
Half-life^a^	40 h	12–17 h	5–9 h	9–14 h	10–14 h
Renal elimination	None	80 %	33 %	25 %	35–50 %
Protein binding (%)	>99	35	90	87	55
Dialyzable	No	Yes	No	No	Possible
Interactions	Many	P-gp	3A4, P-gp	3A4, P-gp	P-gp
Coagulation monitoring	Yes	No	No	No	No
Antidote	Vitamin K	Idarucizumab	No	No	No
Lab measure	INR	aPTT TT, ECT	PT Anti-Xa	Anti-Xa	Anti-Xa

^a^Normal renal function

*P-gp* P glycoprotein, *3A4* cytochrome P450 3A4, *INR* international normalized ratio, *PT* prothrombin time, *aPTT* activated partial thromboplastin time, *TT* thrombin time, *dTT* dilute thrombin time, *ECT* ecarin clotting time

**Fig. 3:**
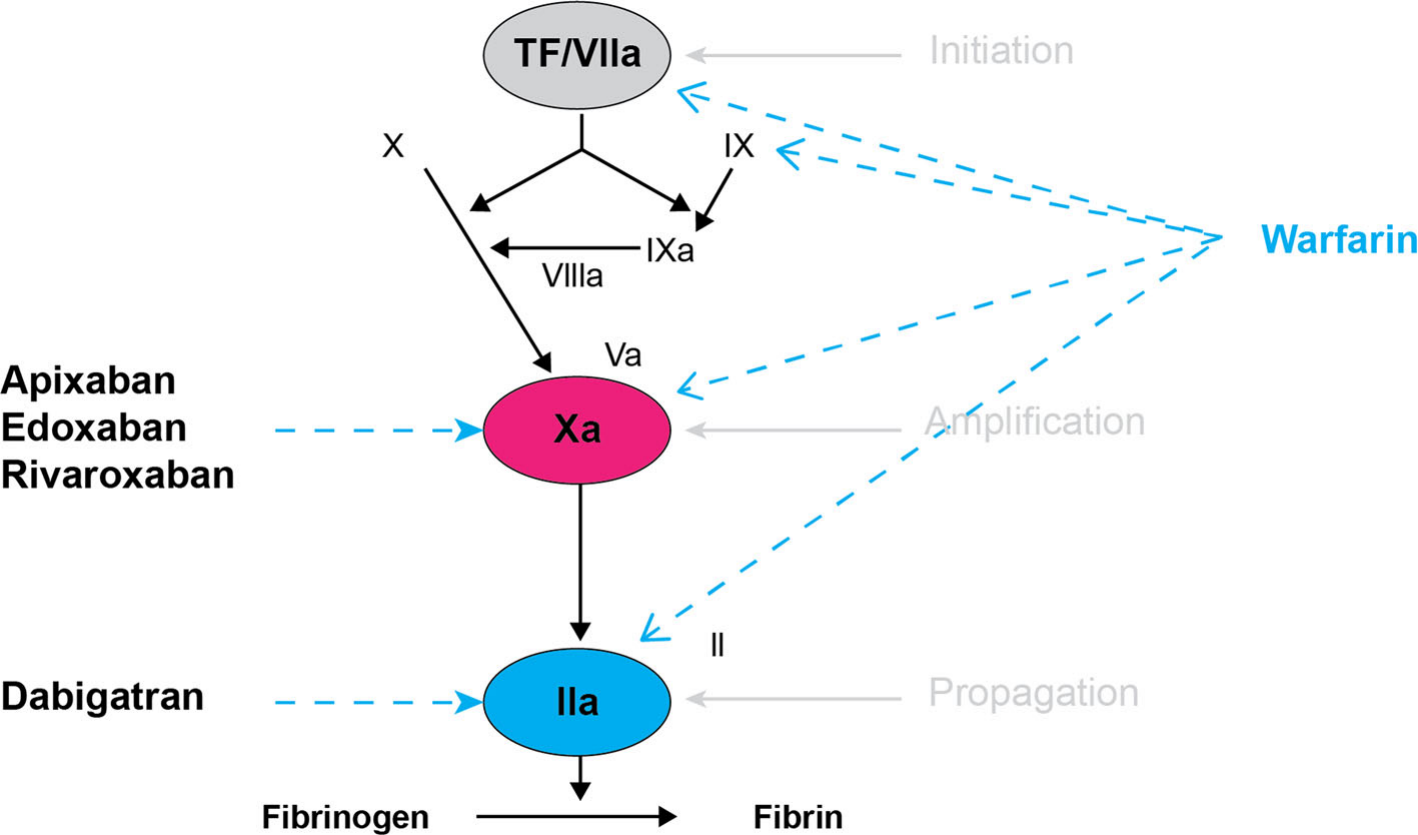
Mechanism of action of the direct oral anticoagulants. Reprinted with permission from: J Thromb Haemost 2005;3:1843–53

The pharmacokinetic and pharmacodynamics properties of DOACs are considerably different than those of warfarin and will be discussed in detail below (Table 6; Fig. 4).

**Fig. 4:**
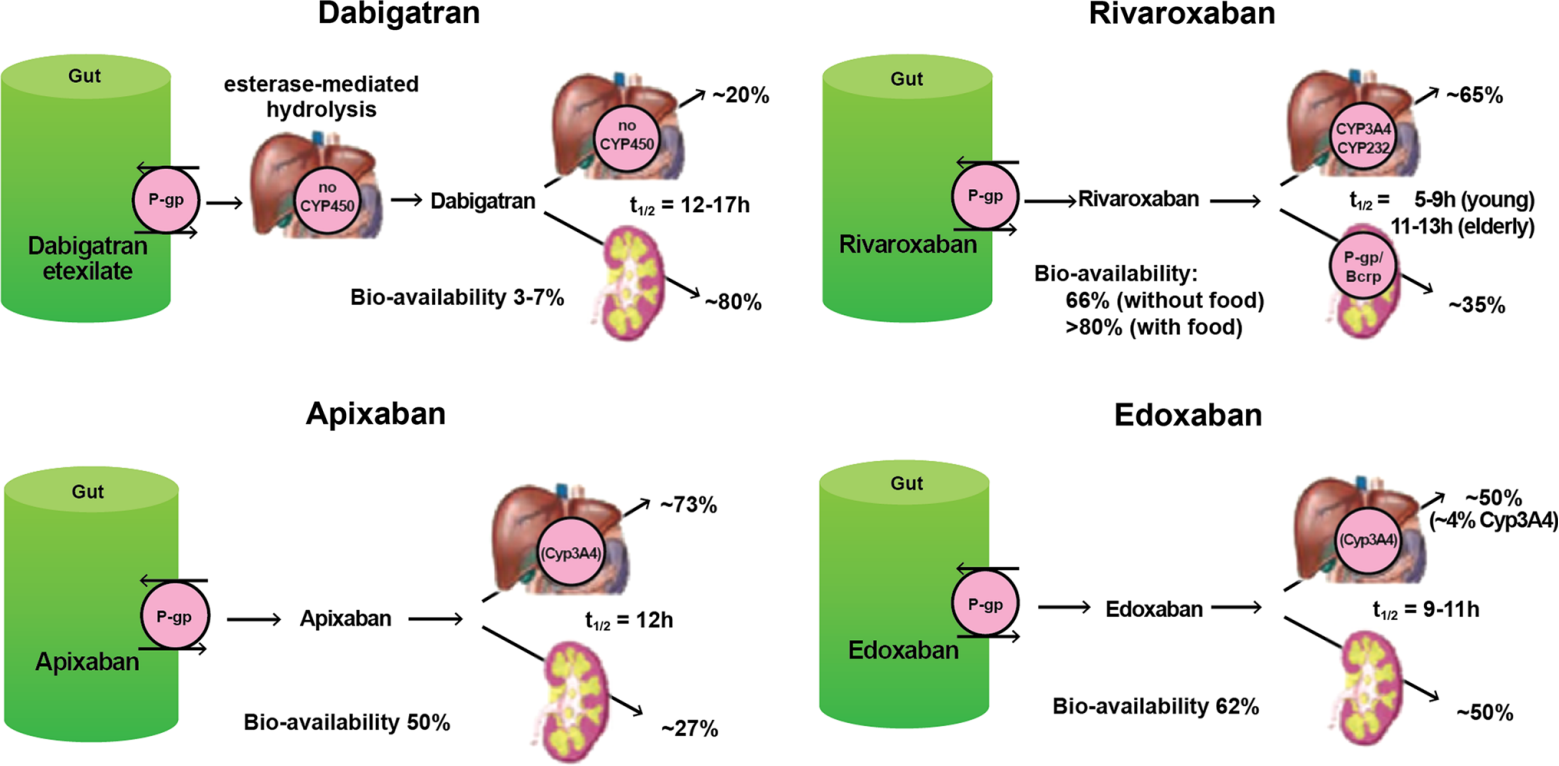
Routes of absorption, metabolism and elimination of the direct oral anticoagulants. Reprinted with permission from [68]

### Direct thrombin inhibitors (DTIs)

#### Dabigatran etexilate

Dabigatran is a potent, competitive DTI that reversibly and specifically binds both clot-bound and free thrombin, as well as inhibiting thrombin-induced platelet aggregation. (Fig. 3) It is a highly polarized, hydrophilic molecule that is not absorbed after oral administration. The commercial product is formulated as a lipophilic prodrug, dabigatran etexilate, to promote gastrointestinal absorption prior to metabolism to the active drug, dabigatran [69].

Dabigatran etexilate has a mean absolute bioavailability of 6.5 % that is independent of dose or dietary intake. Because it requires an acidic environment for maximal dissolution and absorption, pellets containing a tartaric acid core are coated with dabigatran etexilate then placed in capsules for oral administration [69]. The oral bioavailability of dabigatran etexilate increases by 75 % when taken without the capsule shell. The capsules should not crushed, chewed or opened to facilitate administration, as this could lead to excessive exposure to the drug [59]. Gastric acid-suppressing agents can cause minor reductions in exposure to dabigatran, but these reductions are not considered to be clinically relevant [59, 69].

The prodrug dabigatran etexilate is a substrate of the P-glycoprotein (P-gp) efflux system (Fig. 4). Concomitant use of dabigatran and any P-gp *inducer* should be avoided, as this may lead to reduced concentrations (via more rapid absorption and elimination) of dabigatran (Table 7) [59]. Caution or avoidance of dabigatran should be exercised with any concomitant P-gp *inhibitor*, particularly in the setting of moderate to severe renal dysfunction, as this may lead to accumulation of dabigatran [59].

**Table 7:** P-gp inhibitors and inducers (list is not exhaustive): adapted from [57, 70]

P-gp inhibitors			P-gp inducers
Amiodarone	Grapefruit	Propafenone	Barbiturates
Carvedilol	Indinavir	Quinidine	Carbamazepine
Clarithromycin	Itraconazole	Ritonavir	Dexamethasone
Conivaptan	Ketoconazole	Saquinavir	Phenytoin
Cyclosporine	Lepatinib	Tacrolimus	Rifampin
Diltiazem	Mefloquine	Tamoxifen	St. John’s Wort
Dronaderone	Nelfinavir	Verapamil	
Erythromycin	Nicardipine		

Once absorbed, dabigatran etexilate rapidly undergoes esterase-catalyzed hydrolysis and is metabolized to its active form, dabigatran. Dabigatran is 35 % protein bound and has a moderate volume of distribution (V_d_) of 50–70 L [69]. Peak plasma concentrations (C_max_) are achieved within 1.5–3 h (Table 6). Steady state concentrations are achieved approximately 3 days after multiple dose administration, with no evidence of significant accumulation.

Dabigatran is not a substrate of the hepatic CYP P450 isoenzyme system [69]. Renal excretion of unchanged dabigatran is the predominant pathway for elimination, accounting for 80 % of its total clearance. The remainder of the drug undergoes conjugation to form acyl glucuronides that are hepatically eliminated. The elimination t_1/2_ is 12–17 h, independent of dose, in healthy volunteers. In patients with moderate renal impairment (CrCl ≥ 30–50 mL/min) exposed to dabigatran, the AUC increases 3.2-fold and the t_1/2_ increases to 18 h compared to 14 h in healthy subjects. Among patients with severe renal impairment (CrCl 15–30 mL/min), there is a 6.3-fold increase in AUC and the t_1/2_ of dabigatran increases to almost 28 h [71]. Subjects with severe liver disease were excluded from clinical trials of dabigatran. In those with moderate hepatic impairment (Child-Pugh B), the pharmacokinetic profile of dabigatran is not affected. Gender, age, race or extremes of weight (<50 or >110 kg) do not significantly impact dabigatran pharmacology [69].

The aPTT will typically be prolonged in a patient who has recently taken dabigatran [67, 72]. However, a normal aPTT does not exclude clinically relevant dabigatran activity and a prolonged aPTT may underestimate supratherapeutic dabigatran levels [67, 73]. If it is necessary to confirm absence of even minute dabigatran concentrations, use of the more sensitive, undiluted thrombin time (TT) is suggested. To estimate the plasma concentration (and the magnitude of anticoagulant effect present), use of the dilute thrombin time (dTT) or ecarin-based assays should be considered if they are available. The PT and the INR should not be used to measure dabigatran due to insensitivity, significant variation between reagents and lack of standardization across laboratories [67, 72, 73].

### Factor Xa inhibitors

The Factor-Xa inhibitors, apixaban, rivaroxaban and edoxaban, share a similar mechanism of action. They are all competitive, selective and potent direct Factor-Xa inhibitors that bind in a reversible manner to the active site of both free-floating Factor-Xa and Factor-Xa within the prothrombinase complex, thereby attenuating thrombin generation (Fig. 3). These agents are not prodrugs and do not require activation.

#### Apixaban

Apixaban has an absolute oral bioavailability of 50 %, is quickly absorbed in the stomach and small intestine and reaches C_max_ at 1–3 h (Table 6). It is highly protein bound (87 %) and has a small volume of distribution (21–23 L). Following multiple daily doses, steady state concentrations are reached by day 3 with mild accumulation [74]. Food intake does not affect apixaban [75]. An apixaban 5 mg tablet crushed and suspended in 60 mL of 5 % dextrose in water (D5W) and delivered via nasogastric tube provides similar exposure to that seen in healthy volunteers following a single oral dose of 5 mg apixaban. No data is available for crushed or suspended apixaban tablets delivered by mouth [60].

Because it is a substrate of both the CYP 3A4/5 hepatic isoenzyme system and P-gp efflux transporter system [76] (Fig. 4), apixaban may be subject to a number of drug interactions. In patients on doses >2.5 mg twice daily, co-administration with strong dual inhibitors of CYP 3A4/5, such as azole antifungals, macrolide antibiotics and protease inhibitors an empiric dose reduction of 50 % has been suggested by the manufacturer in the absence of data. In patients on doses of 2.5 mg twice daily, co-administration with strong dual CYP 3A4/5 and P-gp inhibitors should be avoided. Strong dual inducers of CYP 3A4/5 and P-gp, (e.g., phenytoin, St. John’s Wort, carbamazepine and rifampin) may significantly reduce apixaban exposure, and co-administration of these agents with apixaban should be avoided. (See Table 8 for lists of dual P-gp and CYP 3A4/5 inducers and inhibitors).

**Table 8:** Dual P-gp and CYP 3A4 inhibitors and inducers (list is not exhaustive): adapted from [57, 70]

Dual P-gp and CYP 3A4 inhibitors		Dual P-gp and CYP 3A4 inducers
Clarithromycin	Ketoconazole	Barbiturates
Conivaptan	Nelfinavir	Carbamazepine
Cyclosporine	Posaconazole	Phenytoin
Diltiazem	Ritonavir	Rifampin
Dronaderone	Saquinavir	St. John’s Wort
Grapefruit	Tamoxifen	
Indinavir	Verapamil	
Itraconazole		

Apixaban has no active circulating metabolites and the parent compound constitutes the major drug-related component in plasma, urine and feces [60, 76]. Apixaban has dual pathways of elimination, with approximately 27 % cleared renally and the remainder eliminated via the fecal route (Fig. 4). The terminal elimination t_1/2_ is approximately 8–14 h in subjects with normal renal function. While renal function has no impact on apixaban C_max_, the AUC is increased by 16, 29 and 44 % in patients with mild (CrCl 51–80 mL/min), moderate (CrCl 30–50 mL/min) and severe (CrCl 15–29 mL/min) renal impairment respectively, as compared to subjects with normal renal function. Despite this, no renal dose adjustments are recommended for VTE treatment with apixaban. In a small study (n = 16) of patients with end-stage renal disease (ESRD) on hemodialysis, a single 5 mg dose of apixaban given 2 h before a 4-h hemodialysis session resulted in a 17 % increase in AUC compared to healthy subjects. A 14 % reduction in exposure to apixaban was seen with hemodialysis compared to the off-dialysis period [77]. The results from this single dose study were extrapolated by the manufacturer to suggest that no change in apixaban dose or frequency of dosing is necessary in ESRD patients on HD. It is important to note that these manufacturer recommendations did not take into consideration the impact of multiple doses on apixaban clearance and its potential accumulation.

Low weight patients (<50 kg) have a 20–30 % increased exposure to apixaban compared to normal weight subjects whereas patients >120 kg have a 23–30 % lower exposure to apixaban compared to normal weight subjects [78, 79]. Additionally, elderly patients have a 32 % increase in AUC compared to younger subjects. Apixaban pharmacokinetics are not significantly altered in patients with mild (Child Pugh A) to moderate (Child Pugh B) hepatic impairment [80]. Apixaban has not been studied in patients with severe hepatic impairment and therefore is not recommended for use in these patients [60]. Gender and race do not appear to have clinically relevant influence on apixaban exposure [78, 79].

The chromogenic anti-Factor-Xa assay, calibrated to apixaban, may be used to quantitatively assess for clinically relevant apixaban levels. A standard chromogenic anti-Factor-Xa assay calibrated to UFH or LMWH may be used in a qualitative manner. The aPTT, PT and the INR should not be used to measure apixaban due to insensitivity, significant variation between reagents and lack of standardization across laboratories [67].

#### Rivaroxaban

The bioavailability of rivaroxaban is dose-dependent (Table 6). At a dose of 10 mg, the bioavailability is 80–100 % and may be taken without regard for food. At higher doses, the bioavailability is approximately 66 % in the fasted state, which is increased to >80 % by food intake. Thus, rivaroxaban 15 and 20 mg tablets should be taken with the largest meal of the day [62, 81]. Rivaroxaban is rapidly absorbed and reaches C_max_ 2–4 h after administration. Crushed 15 and 20 mg tablets delivered in applesauce or suspended in 50 mL of water and given via nasogastric tube provide similar drug exposure as an orally administered tablet. Rivaroxaban is highly protein bound (92–95 %) and has a moderate volume of distribution of 50 L [62].

Approximately two thirds of an administered dose of rivaroxaban undergoes biotransformation to inactive metabolites. It is subject to oxidative degradation via CYP 3A4/5 and to a lesser extent CYP 2J2, as well as non-CYP mediated hydrolysis [62]. Like apixaban, rivaroxaban may be affected by medications that are inhibitors or inducers of the CYP enzymatic pathway [81] (Fig. 4).

Rivaroxaban has a dual mode of elimination, with approximately 36 % of the drug excreted unchanged in the urine, and the remaining two thirds (in the form of inactive metabolites) excreted fairly equally between the renal and hepatobiliary route. Rivaroxaban is a P-gp substrate, not only at the level of gut absorption, but also at the level of elimination in the kidney. Medications that are inhibitors or inducers of the p-glycoprotein may impact plasma concentrations of rivaroxaban via changes in both absorption and elimination (Fig. 4). The terminal elimination t_1/2_ of rivaroxaban is 5–9 h in young healthy subjects, and increases to 11–13 in the elderly, likely due to age-related decline in renal function [62, 81].

Gender, race, age and extremes of weight (<50 kg or >120 kg) do not significantly impact the pharmacokinetics or pharmacodynamics of rivaroxaban. In patients with mild (CrCl 50–79 mL/min), moderate (CrCl 30–49 mL/min) and severe (CrCl <30 mL/min) renal dysfunction, the C_max_ of rivaroxaban is unaffected, but AUC increases by 44, 52 and 65 %, respectively compared to healthy subjects [81]. Mild hepatic impairment has minimal impact on the pharmacokinetics and pharmacodynamics of rivaroxaban [82]. Patients with moderate hepatic impairment (Child-Pugh B) have significantly increased exposure to rivaroxaban (AUC increased by 127 %) compared to healthy subjects. Patients with severe liver disease have not been studied [62, 82].

Rivaroxaban should not be used concomitantly with medications that are dual P-gp and strong CYP 3A4 inhibitors or inducers (See Table 8). Use with weaker combined P-gp and CYP 3A4 substrates should be undertaken with caution or avoided if possible. Pharmacodynamic studies of rivaroxaban have shown that concomitant use with naproxen, aspirin and clopidogrel increases bleeding times [81].

The chromogenic anti-Factor-Xa assay, calibrated to rivaroxaban, may be used to quantitatively assess for clinically relevant rivaroxaban levels [62, 67]. A standard chromogenic anti-Factor-Xa assay calibrated to UFH or LMWH may be used in a qualitative manner. If a chromogenic anti-Factor-Xa assay is not available the PT may be used to qualitatively measure rivaroxaban, albeit with less sensitivity and linearity. Clinicians should be aware that a normal PT does not exclude clinically relevant rivaroxaban concentrations. The aPTT and the INR should not be used to measure rivaroxaban due to insensitivity, significant variation between reagents and lack of standardization across laboratories.

#### Edoxaban

The absolute oral bioavailability of edoxaban in healthy subjects is 62 % and is not affected by food or dose. It is rapidly absorbed and reaches C_max_ in 1–2 h (Table 6). It is approximately 55 % protein bound and has a large V_d_ > 300 L [64, 83, 84]. Edoxaban undergoes biotransformation to various metabolites, the majority of which are formed via hydrolysis. CYP450 isoenzymes do not have a significant role in the metabolism of edoxaban, with less than 4 % of parent compound being transformed by this pathway [85]. The majority of a dose of edoxaban (70 %) is eliminated as unchanged drug. It has dual mode of elimination, with one third eliminated in the urine and two thirds eliminated in the feces (Fig. 4). The elimination t_1/2_ of edoxaban is 10–14 h in healthy subjects. Edoxaban is a substrate of the P-gp transport system and plasma concentrations may be altered by inhibition or induction of this pathway.

Pharmacokinetic studies suggest that dose-adjusted edoxaban 15 mg daily may provide a viable regimen in patients with severe renal impairment [86] or with end-stage renal disease with or without hemodialysis [87]. Published data on the effect of age, gender, race and extremes of weight are not available.

Concomitant use of edoxaban and strong P-gp inhibitors (e.g. quinidine, verapamil, dronaderone) has been shown to increase exposure to edoxaban by >1.5-fold [64, 83, 88]. Until more is known, concomitant use of edoxaban and P-gp inhibitors or inducers should generally be avoided (Table 7). In the setting of VTE, when edoxaban is used concomitantly with a P-gp inhibitor, the dose should be reduced from 60 mg to 30 mg daily. The administration of edoxaban with either aspirin (both low dose and high dose) and naproxen led to a twofold increase in bleeding time [88].

Limited evidence is available to provide guidance on measurement of edoxaban. Use of the chromogenic anti-Factor-Xa assay, either calibrated to edoxaban, or a standard chromogenic anti-Factor-Xa assay calibrated to UFH or LMWH, to assess for the presence or absence clinically relevant edoxaban effect may be considered. The aPTT, PT and the INR should not be used to measure edoxaban due to lack of evidence and presumed insensitivity, significant variation between reagents and lack of standardization across laboratories that is seen with other direct anti-Xa inhibitors [89].

## Conclusions

Anticoagulant drugs are the foundation of therapy for patients with VTE. While effective, they can also result in hemorrhage and other side effects. Anticoagulant selection should be guided by the risks, benefits and pharmacologic characteristics of each agent for each patient. Their safe use requires not only an in-depth knowledge of their pharmacologic properties but also a comprehensive approach to patient management and education.
